# Efficacy and safety of mesenchymal stem cells in the treatment of systemic sclerosis: a systematic review and meta-analysis

**DOI:** 10.1186/s13287-022-02786-3

**Published:** 2022-03-21

**Authors:** Jiehan Cui, Lu Jin, Meng Ding, Jingjing He, Lin Yang, Shaoxin Cui, Xiaoping Wang, Jun Ma, Aijing Liu

**Affiliations:** 1 Department of Rheumatology and Immunology, the Second Hospital of Hebei Medical University, Shijiazhuang, 050000 Hebei Province China; 2 Hebei Medical University, Shijiazhuang, 050000 Hebei Province China; 3 Hebei Research Center for Stem Cell Medical Translational Engineering, Shijiazhuang, 050000 Hebei Province China; 4 Department of Anatomy, Hebei Medical University, Shijiazhuang, 050000 Hebei Province China; 5 Hebei Key Laboratory of Laboratory Medicine, the Second Hospital of Hebei Medical University, Shijiazhuang, 050000 Hebei Province China

**Keywords:** Mesenchymal stem cells, Systemic sclerosis, Meta-analysis, Treatment

## Abstract

**Background:**

Systemic sclerosis (SSc) is an autoimmune disease with high morbidity and mortality characterized by fibrosis of the skin and internal organs. Some studies have investigated the use of stem cells to treat SSc. Herein, a systematic review and meta-analysis was conducted to determine the efficacy and safety of mesenchymal stem cells (MSCs) in the treatment of SSc.

**Methods:**

PubMed, Embase, Cochrane Library, Web of Science, OVID, China National Knowledge Infrastructure and Wanfang databases were searched up to February 1, 2021. Literature screening, data extraction and quality assessment were conducted independently by two researchers in according to the inclusion and exclusion criteria. The discrepancies were resolved by a third researcher.

**Results:**

A total of 9 studies encompassing 133 SSc patients were included in the study. Compared to the baseline after treatment with MSCs: 1. The modified Rodnan skin score (mRSS) was significantly reduced in patients with SSc (*P* < 0.00001). 2. MSCs decreased the number of digital ulcer, mouth handicap scale, and visual analog scale of hand pain in SSc patients (*P* = 0.0007 and *P* = 0.03, respectively). 3. No statistical differences were detected in Raynaud's condition score and Cochin hand function scale score at 6 months of MSCs therapy (*P* = 0.5 and *P* = 0.62). 4. After 12 months of follow-up, MSCs improve carbon monoxide diffusing capacity and forced vital capacity of SSc patients (*P* < 0.05). 5. Overall, MSCs application was safe; a few cases exhibited swelling at the injection site, diarrhea and arthralgia, which had self-recovery, and no severe adverse events occurred in the included trials.

**Conclusions:**

MSC therapy improves the degree of skin thickening, lung function, and mouth opening and relieves finger ulcers and pain in patients with SSc without severe adverse events. Thus, MSCs or MSCs combined with plasma and traditional medicine might be an effective and promising treatment of SSc patients.

*PROSPERO registration number*: CRD42020200350

## Background

Systemic sclerosis (SSc), also known as scleroderma, is an autoimmune disease with high morbidity and mortality characterized by the fibrosis of skin and internal organs, including the heart, lungs, kidneys and digestive tract [1]. The pathogenesis of SSc is complex and has not yet been elucidated. Presently, the main therapy of SSc is symptomatic treatment. Glucocorticoid and immunosuppressive agents fail to shorten the course of the disease and improve prognosis, and patients might suffer adverse reactions from the drugs. Currently, patients with SSc are in the spotlight during the period of coronavirus disease 2019(COVID-19) because of the co-morbidity of interstitial lung disease (ILD) and high risk of developing pneumonia with the widespread use of immunosuppressive agents [2]. Once infected, the patients have to face the risk of disease deterioration and even death.

Stem cells play a key role in tissue homeostasis, repair, and regeneration. Also, autologous hematopoietic stem cell transplantation (AHSCT) has been included in the updated European League Against Rheumatism (EULAR) guidelines for treatment of rapidly progressing SSc [3]. Mesenchymal stem cells (MSCs) were discovered in 1976 as a fibroblast-like cell population capable of generating osteogenic precursors [4]. These cells maintain their multidirectional differentiation potential [5]. Currently, MSCs are widely used in many fields such as autoimmune diseases, neurological diseases, endocrine diseases and orthopedic diseases [6–10]. Recent studies have shown that MSCs can be used as a potential therapeutic tool for COVID-19 [10–13]. They could inhibit the proliferation and function of immune cells, including T cells and B cells, through paracrine mechanism via a series of soluble factors [14]. These immune cells play a key role in host defense against viral infection and immune surveillance against cancer [14]. However, the applications of MSCs in patients with SSc are yet controversial. Herein, we aimed to carry out a systematic literature review and meta-analysis of all the published data to evaluate the efficacy and safety of MSCs in the treatment of patients with SSc and provide evidence for clinical application.

## Methods and analysis

### Patient and public involvement statement

There were no patient or public involved in this systematic review and meta-analysis. No patient was asked to advise on interpretation or write the results.

### Study design

This study was prospectively registered on PROSPERO (CRD42020200350) and performed in accordance with the Preferred Reporting Items for Systematic Reviews and Meta-analyses (PRISMA) guidelines [15]. The body of evidence was assessed by grading of recommendations, assessment, development and evaluation (GRADE) approach [16].

Type of research: Clinical trials.

Type of participants and interventions:

1. Inclusion criteria: SSc adults treated with MSCs (as diagnosed by a clinician, or using any recognized diagnostic criteria) were included, regardless of age, gender, disease duration and severity.
2. Exclusion criteria were as follows: (1) Non-Chinese or English literatures; (2) Repeatedly published data; (3) Literature with incomplete data or lacked target indicators; (4) Non-clinical studies such as animal-based, review articles, case reports, conference reports, replies, patents or protocols.

### Outcome assessment

The effective outcome endpoints include at least one of the six aspects of the modified parameters of disease activity in SSc patients based on the EULAR scleroderma trial study group: recent skin changes, digital ulcer (DU), modified Rodnan skin score (mRSS), tendon friction rub, C-reactive protein (CRP), and diffusing capacity of the lung for carbon monoxide (DLco) in SSc patients [17]. In addition, mouth handicap in systemic sclerosis scale (MHISS), Cochin hand function scale (CHFS) and visual analog scale (VAS) for hand pain were included. Adverse events (AEs) were selected as safety outcome measures.

Search methods for identifying relevant trials.

### Search strategy

A comprehensive literature search was performed to identify the relevant publications in PubMed, Embase, Cochrane Library databases, Web of Science, OVID, China National Knowledge Infrastructure and Wanfang databases from their inception to February 1, 2021. The search strategies typically use a combination of terms from medical subject headings (MeSH) and free-text keywords. The English subject headings were “scleroderma, systemic” AND “mesenchymal stem cells”, combined with free words as follows: (systemic sclerosis OR scleroderma, diffuse OR scleroderma, progressive OR CREST syndrome) AND (mesenchymal stromal cells OR MSC OR multipotent stromal cells OR mesenchymal progenitor cells OR Wharton jelly cells OR adipose-derived mesenchymal stem cells OR bone marrow stromal stem cells). The Chinese subject headings are “硬皮病, 系统性”AND “间充质干细胞”, respectively. The combination of (系统性硬化症 OR 硬皮病 OR 局限性硬皮病 OR 弥漫性硬皮病 OR 重叠综合征 OR 进行性全身硬化症) AND(间充质干细胞移植 OR 间质干细胞 OR 脂肪间充质干细胞 OR 骨髓间充质干细胞 OR 脐带间充质干细胞) was searched as well. Manual search and other methods were supplemented to reduce the missed detection rate.

### Screening

The literature retrieved from each database was imported into the EndNote reference manager, and the duplicated articles were removed. Then, titles, abstracts and full texts were scrutinized to determine the eligible studies after excluding the irrelevant articles by two investigators (CJH/JL) independently. Any disagreements were resolved by a consensus. Any discrepancies were addressed by a third researcher (DM) who decided on the final results. In the case of unavailability of full-text, the data were obtained by contacting the original author through telephone or e-mail.

### Data extraction and quality assessment

Two investigators (CJH/CSX) independently extracted the data, including post-treatment outcomes, standard deviations, and the number of participants in each group. Then the data of the first author/published year, country, research type, number of cases (female/male), mean age, follow-up time, cell type and number of MSCs, injection method, and endpoint index were extracted. The risk of bias of the included randomized controlled trials (RCT) was evaluated using the Cochrane risk of bias assessment tool, and the risk of bias of non-RCT studies was evaluated using the Newcastle–Ottawa scale(NOS) [18], with respect to the population selection, intergroup comparability and result evaluation with a total score of 9 points (≤ 4 points is low quality, 4–7 points is medium quality and ≥ 7 points is high quality). Next, we assessed the certainty of evidence using GRADE framework [16]. The two investigators (CJH/CSX) mutually cross-checked the included literature and conducted a quality assessment, and the third investigator (HJJ) decided the final result in case of any difference. The certainty of the evidence was then classified as high, moderate, low, or very low [16]. High certainty indicated that we are very confident that the true effect lies close to that of the estimate of the effect. Moderate certainty meant that we are moderately confident in the effect estimate; the true effect is likely to be close to the estimate of the effect, but there is a possibility of substantial difference. Low certainty meant our confidence in the effect estimate is limited; the true effect might be substantially different from the estimate of the effect. Very low certainty indicated that we have very little confidence in the effect estimate; the true effect is likely to be substantially different from the estimate of the effect [16].

### Statistical analysis

The extracted data were pooled and analyzed using the Cochrane Collaboration Software Revman 5.3. For dichotomous data, pooled outcomes were presented as odds ratio (OR) and 95% confidence interval (CI), while continuous outcomes were expressed as a mean difference (MD) and 95% CI for analysis. Heterogeneity was statistically evaluated by *I*^2^ value, indicating low, moderate and high heterogeneity with the thresholds of ≥ 25%, ≥ 50% and ≥ 75%, respectively. Typically, *I*^2^ > 50% indicates substantial heterogeneity. In this study, the fixed-effect model was applied for analysis if trials were homogeneous (*I*^2^ ≤ 50% and *P* > 0.1) and the random-effect model was applied for the meta-analysis if statistical heterogeneity was identified (*I*^2^ > 50% and *P* < 0.1); *P* < 0.05 indicated statistical significance. Sensitivity analyses were also analyzed to test the stability of the pooled results. Publication bias was evaluated by STATA software 15.1, using both Egger's linear regression method and Begg’s rank correlation test.

## Results

### Search results

**Fig. 1:**
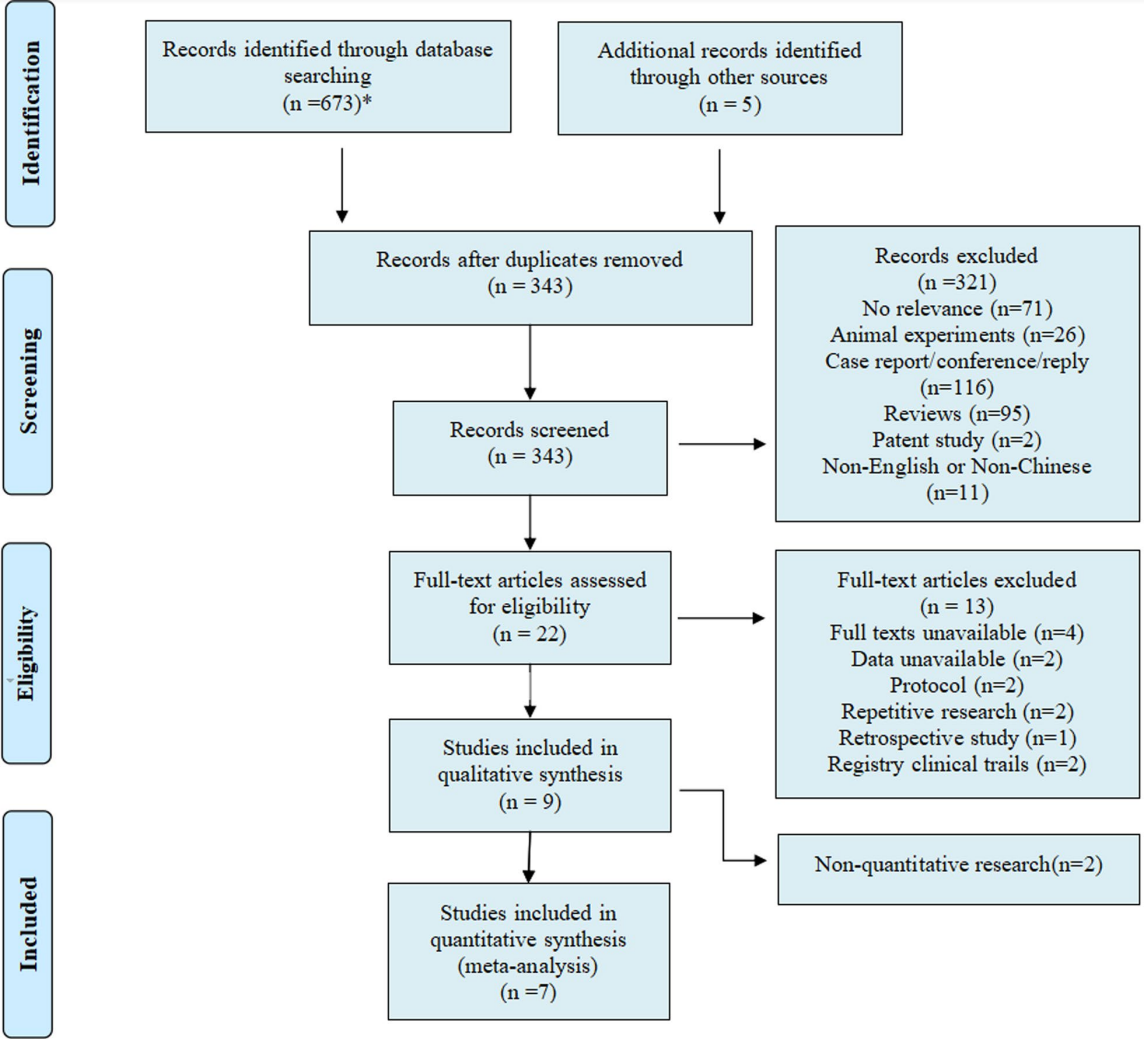
Flow chart of the selection process. Flow chart describing the selection steps of the systematic review and meta-analysis of comparing the efficacy and safety of mesenchymal stem cells in patients with systemic sclerosis, showing the number of studies excluded at each step, as well as the reasons for exclusion. *PubMed (*n* = 100), Ovid (*n* = 98), Cochrane (*n* = 11), Web of Science (*n* = 194), Embase (*n* = 186), Wanfang Data (*n* = 23), and China National Knowledge Infrastructure (*n* = 61). Ultimately, a total of 673 were retrieved from the seven database, and 5 articles were obtained by manual retrieval. Of these 678 studies, nine articles were finally identified, including 7 quantitative studies

According to the above search strategy, 678 articles were initially retrieved from 7 databases, and 343 were obtained after the removal of duplicates. Then the titles and abstracts were screened for potential eligibility, and 11 articles were considered for full-text review which met the inclusion criteria. Of these, 3 were independent studies of the same population with different follow-up durations [19–21]. Finally, 9 studies were identified, including 7 quantitative studies. The specific screening process is illustrated in Fig. 1.

### Study characteristics

The characteristics of included studies are summarized in Table 1. A total of 9 clinical studies, including 133 adult SSc patients, were finally included, all of which were self-controlled studies. These were conducted in 6 countries, among which 2 were in China, 3 in Italy, 1 in the USA, 1 in France, 1 in Germany, and 1 in Korea, reporting on diffuse, localized, progressive, and refractory SSc patients to conventional treatment. The age range of the cohort was 37.4–56 years, and the follow-up time was from 12 weeks to 30 months, and 2 studies were without gender detail, 3 were without the mean age of patients and 1 was without follow-up time. In terms of cell sources, MSCs were derived from adipose-derived mesenchymal stem cells (ADSCs) in 6 included studies, 3 from stromal vascular fraction (SVF) after adipose tissue removal of mature adipocytes), 1 from bone marrow-derived MSCs (BMSCs), and 2 from umbilical cord MSCs (UC-MSCs) with cell numbers 0.7–1.8 × 10^6^/Kg by intravenous or hypodermic injection.

**Table 1:** Basic characteristics of the included studies

References	Year	Country	Design	Sample size (female/male)	Average age	Follow-up time	Source of MSCs	Cell number of MSCs	Administration method
Almadori [22]	2019	America	Self-control	62 (61/1)	56	12.41 ± 8.64 m	ADSCs	NA	IH
Blezien [23]	2017	Italy	Self-control	7 (7/0)	46.28	1/6/12 m	ADSCs	NA	IH
Francesco [24]	2017	Italy	Self-control	6 (4/2)	NA	3 m	SVF	NA	IH
Granel [19–21]	2015	France	Self-control	12 (12/0)	54.5	2/6/12/22/30 m	SVF	NA	IH
Keyszer [25]	2011	Germany	Self-control	3 (1/2)	54.67	3/6 m	BMSCs	0.7–1.8 × 10^6^/kg	IV
Park [26]	2020	Korea	Self-control	18 (15/3)	NA	2/6/24w	SVF	3.61 × 10^6^	IH
Scuderi [27]	2013	Italy	Self-control	6 (4/2)	NA	12 m	ADSCs	8 × 10^5^/ml of HA	IH
Wang [28]	2013	China	Self-control	5 (2/3)	44.6	1/3/6/12 m	UC-MSCs	1 × 10^6^/kg	IV
Zhang [29]	2017	China	Self-control	14 (11/3)	37.4	1/3/6/12 m	UC-MSCs	1 × 10^6^/kg	IV

NA, not applicable; m, month; w, week; HA, hyaluronic acid; IV, intravenous injection; IH, hypodermic injection; ADSCs, adipose derived mesenchymal stem cells; UC-MSCs, umbilical cord mesenchymal stem cells; SVF, stromal vascular fraction; BMSCs, bone marrow mesenchymal stem cells

### Outcome measures and quality evaluation of the included studies

In this study, VAS, MHISS, mRSS, Raynaud’s condition score (RCS), CHFS, and DU were finally selected as effective outcome measures for quantitative analysis in 7 studies. In addition, 2 studies with different evaluation criteria, such as skin wrinkling sensation, skin elasticity, and postoperative satisfaction, and 1 study that assessed the changes in DLco in SSc patients before and after MSC treatment were systematically evaluated without quantitative analysis. Moreover, the safety of MSC treatment in SSc patients was evaluated by screening the original data about AEs in the included literatures (Table 2).

**Table 2:** Outcomes and quality evaluation of the included studies

References	Sample size	Number of AEs	AEs		Endpoint*	Risk of bias-NOS
			Injection site skin reactions	Other		
Almadori [22]	62	1	Skin infection (1)	None	①②	6
Blezien [23]	7	NA	Lip oedema and pain	None	②	8
Francesco [24]	6	NA	None	None	Skin changes	7
Granel [19–21]	12	2	Transient paresthesia of finger (2)	None	①③④⑤	8
Keyszer [25]	3	3	None	Minor respiratory tract infection (3)	③	7
Park [26]	18	4	Transient pale fingers (3)	Dizziness after local anesthesia (1)	①③④⑤⑥	7
Scuderi [27]	6	NA	None	None	Skin changes	5
Wang [28]	5	0	None	None	③⑥	8
Zhang [29]	14	6	None	Minor respiratory tract infection (5)/diarrhea (1)	③⑦	8

^*^①VAS, visual analogue scale; ②MHISS, mouth handicap in systemic sclerosis scale; ③mRSS, modified Rodnan skin score; ④RCS, Raynaud’s condition score; ⑤CHFS, cochin hand function scale score; ⑥DU, digital ulcer; ⑦DLco, carbon monoxide diffusing capacity. NA, not applicable

A total of 9 articles were evaluated using the NOS scale, of which 2 were moderate quality, and 7 were high quality. As shown in Table 3, the evidence was judged to be of low quality for the RCS and MHISS outcomes. For mRSS outcome, evidence ranged from moderate to low, and the outcomes DU, VAS, and CHFS were judged to be very low.

**Table 3:** Summary of findings and certainty of evidence for efficacy

	Summary of findings		Certainty of evidence				Certainty of evidence
	No of participants (No of trials)	Mean difference (95% CI)	Study design	Inconsistency	Imprecision	Small study effects	
*mRSS*							
3 m	22 (3)	4.11 (2.19 to 6.02)	Downgraded*	Not downgraded	Not downgraded	Not downgraded	Moderate
6 m	52 (5)	5.09 (3.38 to 6.81)	Downgraded*	Not downgraded	Not downgraded	Not downgraded	Moderate
12 m	19 (2)	6.49 (4.61 to 8.37)	Downgraded*	Not downgraded	Not downgraded	Not downgraded	Moderate
*mRSS*							
SVF	30 (2)	4.65 (0.74 to 8.57)	Downgraded*	Not downgraded	Not downgraded	Not downgraded	Moderate
UC-MSCs	19 (2)	5.08 (3.10 to 7.05)	Downgraded*	Not downgraded	Not downgraded	Not downgraded	Moderate
BMSCs	3 (1)	6.70 (− 0.22 to 13.62)	Downgraded*	Not downgraded	Not downgraded	Downgraded^c^	Low
*RCS*							
6 m	30 (2)	1.80(− 3.38 to 6.99)	Downgraded*	Downgraded^a^	Not downgraded	Not downgraded	Low
*DU*							
6 m	36 (4)	21.10 (3.63 to 122.56)	Downgraded*	Not downgraded	Downgraded^b^	Downgraded^c^	Very low
*VAS*							
6 m	92 (3)	7.58 (0.55 to 14.60)	Downgraded*	Downgraded^a^	Not downgraded	Downgraded^c^	Very low
*MHISS*							
12 m	69 (2)	5.52 (2.41 to 8.62)	Downgraded*	Downgraded^a^	Not downgraded	Not downgraded	Low
*CHFS*							
6 m	30 (2)	9.05 (− 27.01 to 45.11)	Downgraded*	Downgraded^a^	Downgraded^b^	Downgraded^c^	Very low

mRSS, modified Rodnan skin score; SVF, stromal vascular fraction; UC-MSCs, umbilical cord mesenchymal stem cells; BMSCs, bone marrow-derived mesenchymal stem cells; RCS, Raynaud’s condition score; DU, digital ulcer; VAS, visual analogue scale; CHFS, cochin hand function scale score; MHISS, mouth handicap in systemic sclerosis scale; m, month

*Downgraded by one level because > 25% of participants in this comparison were from studies at high risk of bias

^a^Downgraded by one level because heterogeneity (*I*^2^) > 50%

^b^Downgraded by one level because the limits of the 95% confidence interval were 20 points different to smallest worthwhile effect

^c^Downgraded by one level owing to small study bias

## Skin changes in SSc patients

### mRSS

**Fig. 2:**
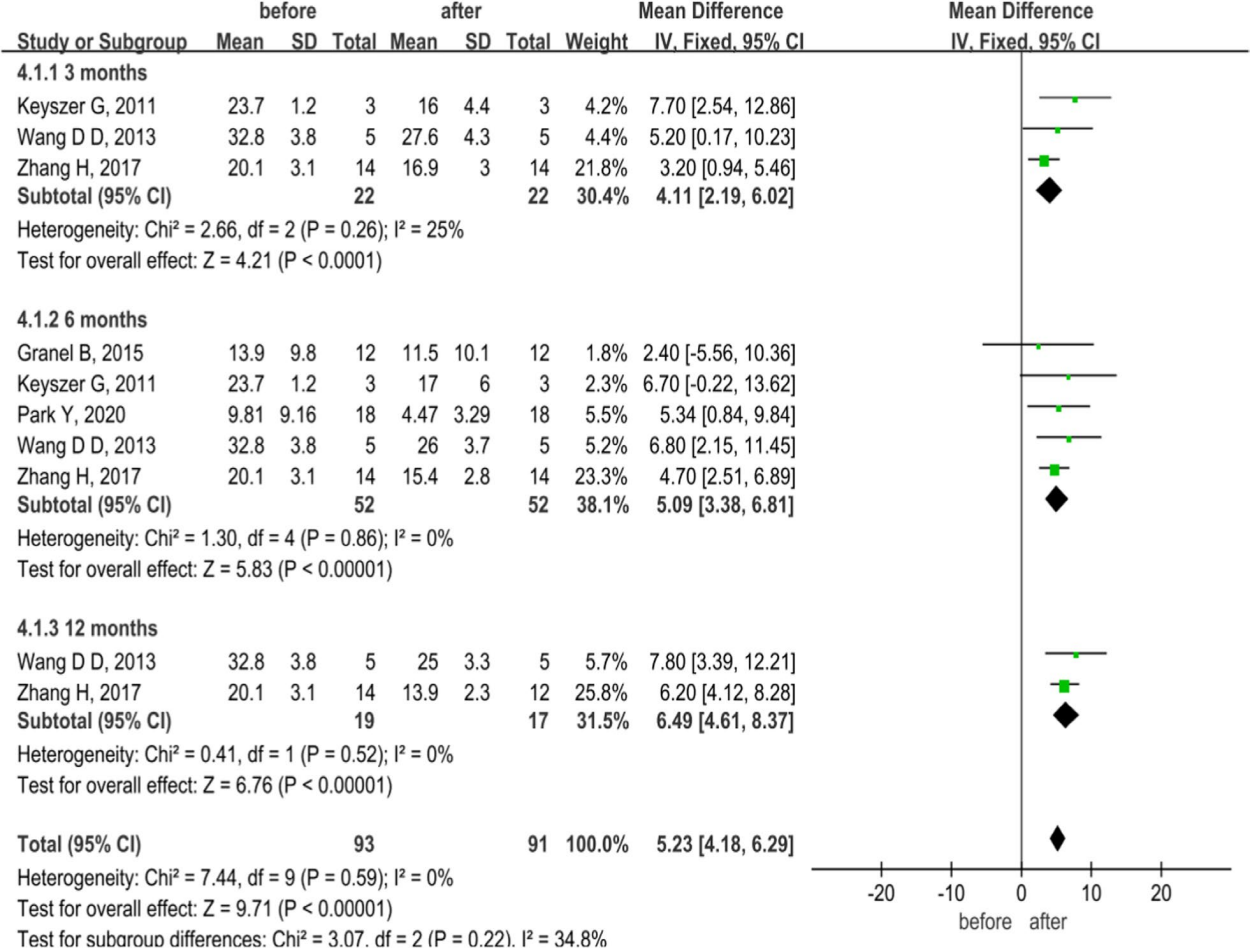
Forest plot of mRSS changes before and after treatment with MSCs at different time points. The mRSS of SSc patients after MSCs treatment was significantly lower than pretreatment at different follow-up times involving 3, 6, and 12 months (*P* < 0.05), especially at 12 months. No significant heterogeneity was observed in any of the three groups, and a fixed-effects model was used for statistical analysis. In the plane rectangular coordinate system, the forest plot takes a vertical invalid line (scale of abscissa is 0) as the center, describes the effect quantity and 95% CI of each study by using multiple line segments parallel to the horizontal axis, and describes the effect quantity and confidence interval of multiple studies by using a diamond. mRSS, modified Rodnan skin score; MSCs, mesenchymal stem cells; CI, confidence interval; SMD, standardized mean difference

A total of 5 studies involving 52 SSc patients were compared using mRSS changes before and after MSC treatment. A fixed-effect model was used for low heterogeneity among all studies and within each subgroup at different follow-up times (*P* > 0.1, *I*^2^ ≤ 50%; Fig. 2). Meta-analyses showed that the mRSS of SSc patients after MSC treatment was significantly lower than pretreatment, suggesting that the degree of skin thickening was significantly reduced (MD = 5.23, 95% CI 4.18–6.29, *P* < 0.00001; Fig. 2).

The subgroup analyses were conducted according to different follow-up times after MSC treatment. The results revealed that MSCs could reduce the mRSS of patients at different follow-up times of 3, 6, and 12 months, respectively (all *P* < 0.0001; Fig. 2), and the forest plot showed that the mRSS at 12 months decreased most significantly.

**Fig. 3:**
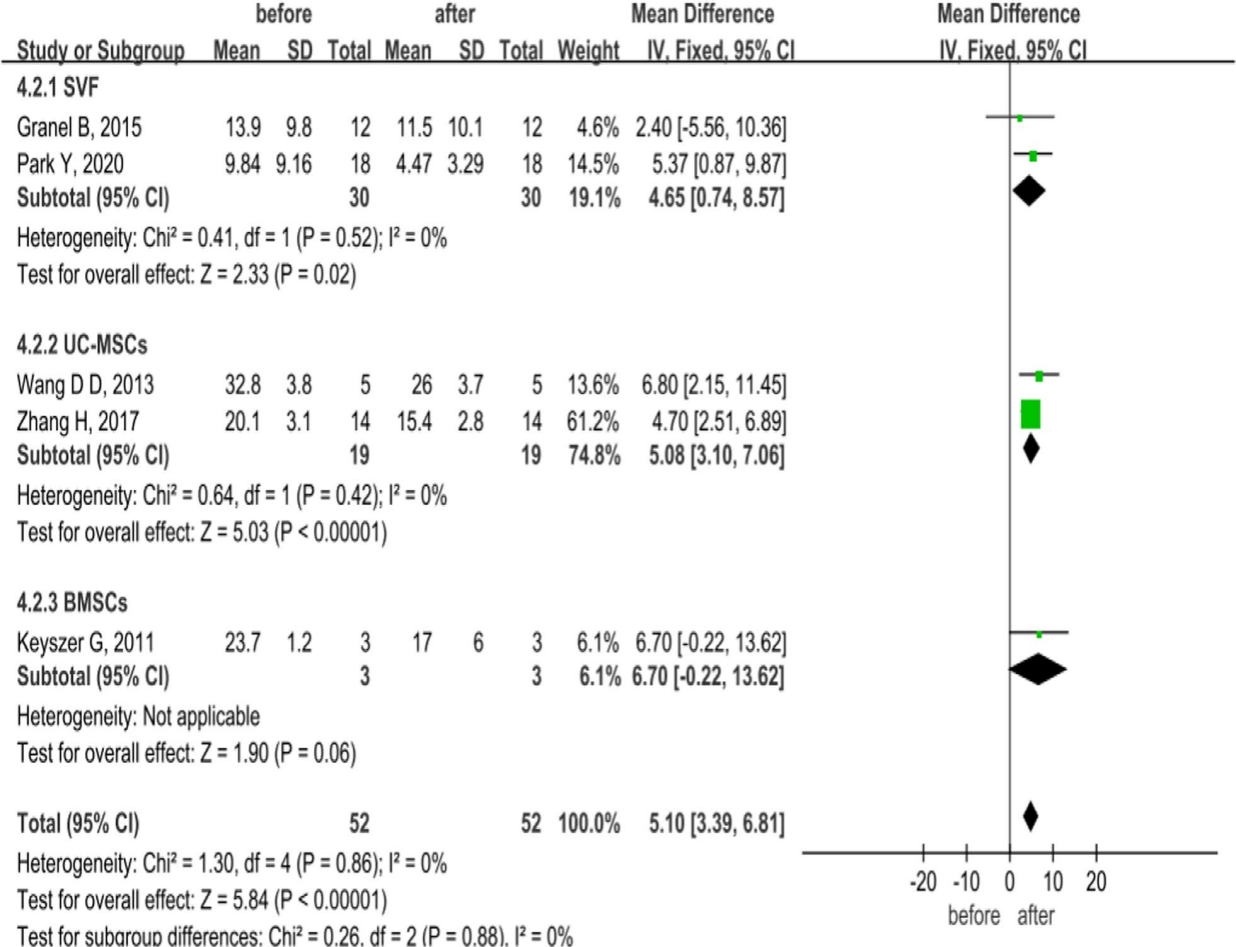
Forest plot of mRSS changes before and after treatment with MSCs of different cell origins. The results showed mRSS score was significantly lower than pretreatment at 6 months of MSCs from different sources treatment (*P* < 0.05). No significant heterogeneity was observed in any of the three groups, and a fixed-effects model was used for statistical analysis. In the plane rectangular coordinate system, the forest plot takes a vertical invalid line (scale of abscissa is 0) as the center, describes the effect quantity and 95% CI of each study by using multiple line segments parallel to the horizontal axis, and describes the effect quantity and confidence interval of multiple studies by using a diamond. SVF, stromal vascular fraction; MSCs, mesenchymal stem cells; UC-MSCs, umbilical cord mesenchymal stem cells; BMSCs, bone marrow-derived mesenchymal stem cells; mRSS, modified Rodnan skin score; CI, confidence interval; SMD, standardized mean difference

In order to evaluate the effect of MSCs from different cell sources on mRSS in SSc patients, the common follow-up time of 6 months was selected for analysis in SVF, UC-MSCs, and BMSCs. Heterogeneity analysis showed homogeneity (*P* = 0.86, *I*^2^ = 0%; Fig. 3), and fixed-effects model analysis indicated that MSCs from different sources improved mRSS and reduced the degree of skin thickening in patients with SSc at 6 months of treatment (MD = 5.10, 95% CI:3.39–6.81, *P* < 0.00001; Fig. 3).

### Skin elasticity and tightness

Francesco et al. [24] demonstrated that increased skin elasticity of cheek and lips in 6 SSc patients 3 months after application of SVF combined with platelet-rich plasma (PRP) via facial injection and raised capillary density at the labial margin in 4 patients. In addition, Scuderi et al. [25] applied ADSCs for local injection in different lesion sites in 6 patients with SSc, and the skin hypopigmentation, elasticity, and sensitivity status of 5 patients were improved after 12 months of treatment compared to those before treatment. Thus, the results of these qualitative analyses suggested that MSCs had some efficacy in improving skin elasticity and tightness.

## Vascular changes in fingers of SSc patients

### RCS

**Fig. 4:**
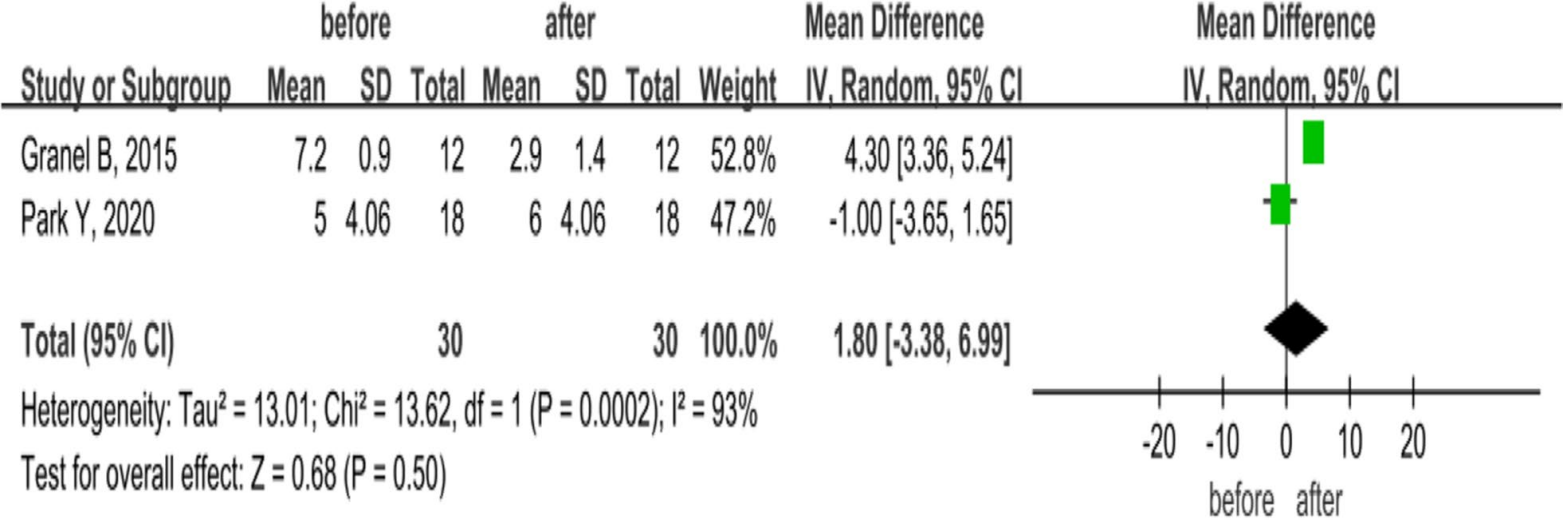
Forest plot of RCS changes before and after treatment with MSCs. Only two studies comparison of RCS value and they were significant heterogeneity (*I*^2^ = 93%). Hence, a random-effects model was used for analysis and no significant difference was observed at 6 months (*P* > 0.05). In the plane rectangular coordinate system, the forest plot takes a vertical invalid line (scale of abscissa is 0) as the center, describes the effect quantity and 95% CI of each study by using multiple line segments parallel to the horizontal axis, and describes the effect quantity and confidence interval of multiple studies by using a diamond. RCS, Raynaud's condition score; MSCs, mesenchymal stem cells; CI, confidence interval; SMD, standardized mean difference

As shown in Fig. 4, 2 studies with 30 SSc patients compared the RCS changes before and 6 months after MSC treatment (local SVF injection). A random-effects model was used for the analysis of consolidated effect values because of the high heterogeneity (*P* = 0.0002, *I*^2^ = 93%), and the results indicated that the RCS of patients did not change significantly (MD = 1.8, 95% CI − 3.38 to 6.99, *P* = 0.50).

### DU

**Fig. 5:**
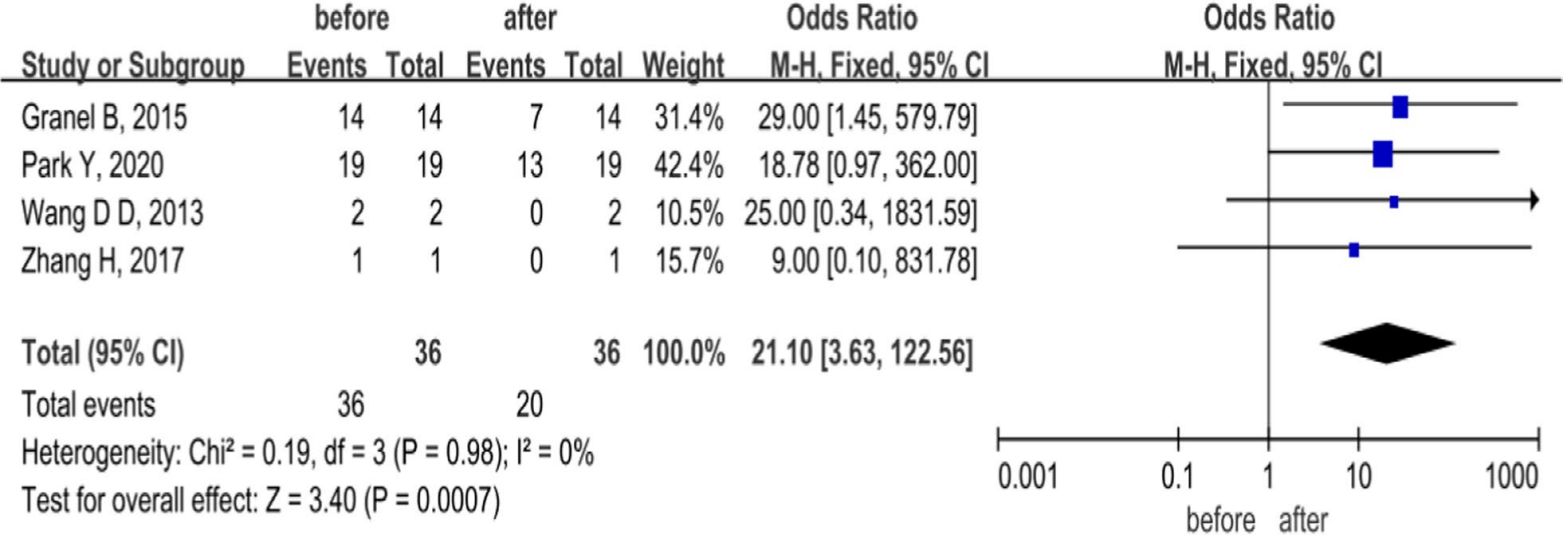
Forest plot of DU changes before and after treatment with MSCs. Four studies showed after treatment with MSCs, DU numbers in SSc patients were significantly lower than pretreatment at 6 months (*P* < 0.05). No significant heterogeneity was observed, and a fixed-effects model was used for statistical analysis. In the plane rectangular coordinate system, the forest plot takes a vertical invalid line (scale of abscissa is 1) as the center, describes the effect quantity and 95% CI of each study by using multiple line segments parallel to the horizontal axis, and describes the effect quantity and confidence interval of multiple studies by using a diamond. However, the confidence interval was large, which might due to small sample size. DU, digital ulcer; MSCs, mesenchymal stem cells; CI, confidence interval

Herein, 4 studies reported changes in DU numbers at 6 months in 58 SSc patients treated with MSCs. Among these, 2 applied autologous SVF via finger injection, and the others used allogeneic UC-MSCs intravenously. A fixed-effects model analysis with no heterogeneity (*P* = 0.98, *I*^2^ = 0%) showed that the DU numbers in SSc patients were significantly reduced after 6 months of treatment with MSCs (OR = 21.10, 95% CI 3.63–122.56, *P* = 0.0007; Fig. 5), suggesting a repair effect of MSCs on skin ulcers in SSc patients. However, the CI was large, which might be due to the small sample size.

### VAS

**Fig. 6:**
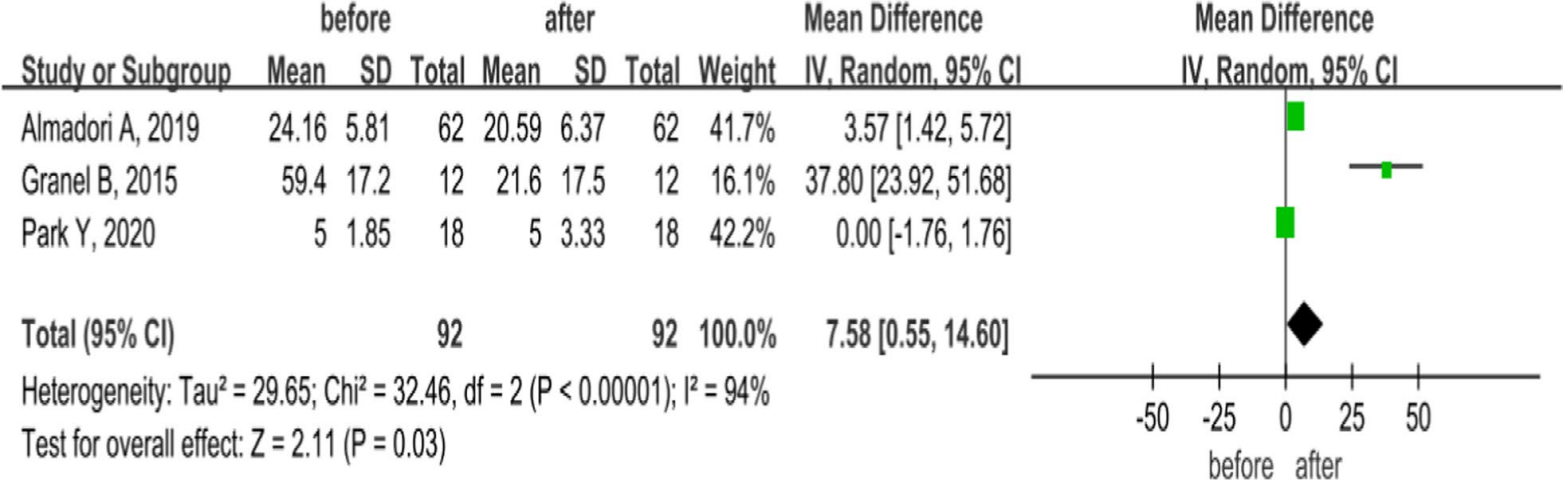
Forest plot of VAS changes before and after treatment with MSCs. Three studies showed after treatment with MSCs, VAS score in SSc patients was significantly lower than pretreatment at 6 months (*P* < 0.05). Since significant heterogeneity was observed (*I*^2^ = 94%), a random-effects model was used. In the plane rectangular coordinate system, the forest plot takes a vertical invalid line (scale of abscissa is 0) as the center, describes the effect quantity and 95% CI of each study by using multiple line segments parallel to the horizontal axis, and describes the effect quantity and confidence interval of multiple studies by using a diamond. VAS, visual analogue scale; MSCs, mesenchymal stem cells; CI, confidence interval; SMD, standardized mean difference

As shown in Fig. 6, 3 included studies compared the VAS of ulcers in SSc patients treated with MSCs for 6 months; of these, 2 applied SVF via finger injection, and 1 study applied ADSCs by facial injection. The results showed that MSC treatment reduces the VAS score in both hands of patients at 6 months (MD = 7.09, 95% CI 0.53–13.65, *P* = 0.03), significantly improving the hand ulcer pain in SSc patients; however, high heterogeneity was observed (*P* < 0.00001, *I*^2^ = 94%). A random-effects model that accounts for statistical heterogeneity between the studies and provides a more conservative estimate of the significance than a fixed-effects model was used. Sensitivity analysis was conducted by eliminating the studies sequentially. No significant changes were observed in combining the results, indicating that the outcomes were stable and reliable.

## Perioral and tendon function

### MHISS

**Fig. 7:**
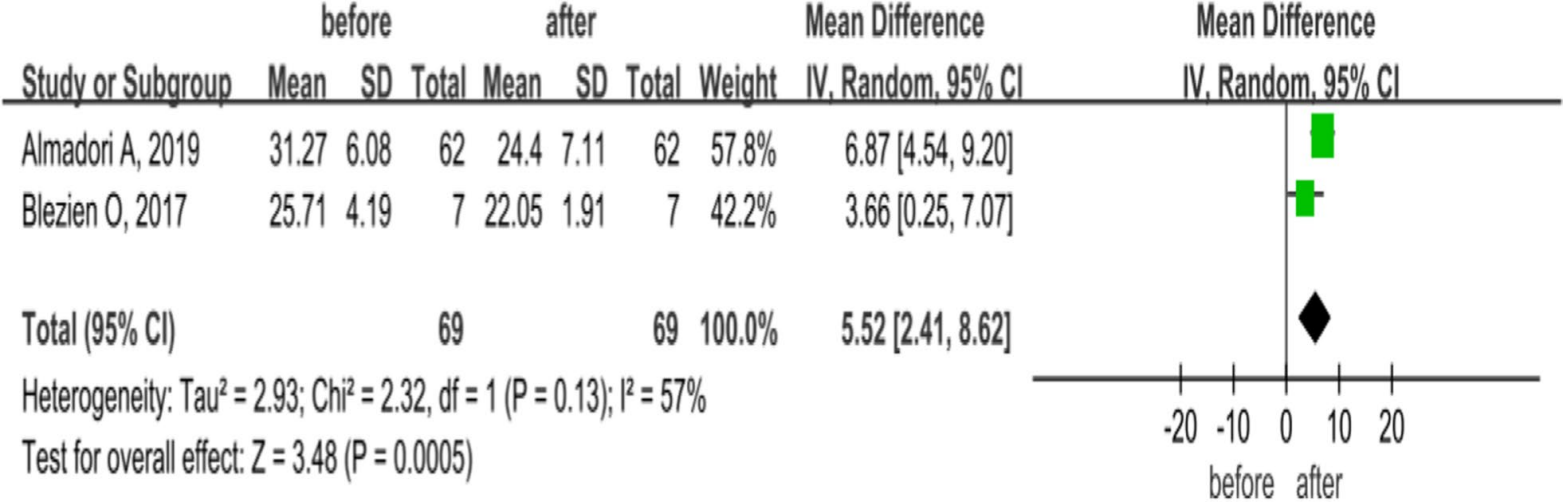
Forest plot of MHISS changes before and after treatment with MSCs. Only two studies showed after treatment with MSCs, MHISS score in SSc patients was significantly lower than pretreatment at 12 months (*P* < 0.05). Since heterogeneity was observed (*I*^2^ = 57%), a random-effects model was used. In the plane rectangular coordinate system, the forest plot takes a vertical invalid line (scale of abscissa is 0) as the center, describes the effect quantity and 95% CI of each study by using multiple line segments parallel to the horizontal axis, and describes the effect quantity and confidence interval of multiple studies by using a diamond. MHISS, mouth handicap in systemic sclerosis scale; MSCs, mesenchymal stem cells; CI, confidence interval; SMD, standardized mean difference

MHISS consists of 12 items, which could effectively evaluate the oral dysfunction in patients with SSc [30, 31]. Each item was scored from 0–4, with a total score of 0 (no disorder) to 48 (severe disorder). The 12 items comprise three aspects: mouth opening, degree of salivary gland involvement, and aesthetic score. Two articles compared the MHISS changes in 69 SSc patients before and after treatment with MSCs and autologous ADSCs via facial injection and were followed up at 12 months. A random-effects model was applied for low heterogeneity (*P* = 0.13, *I*^2^ = 57%), and results showed that ADSC treatment improved mouth opening, oral swallowing, masticatory function and maxillofacial morphology in patients at 12 months (MD = 5.52, 95% CI 2.41–8.62, *P* = 0.0005; Fig. 7).

### CHSF

**Fig. 8:**
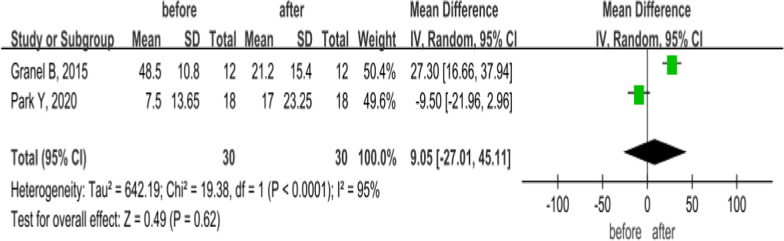
Forest plot of CHFS changes before and after treatment with MSCs. Only two studies showed after treatment with MSCs, CHFS score in SSc patients was significantly lower than pretreatment at 6 months (*P* < 0.05). Since significant heterogeneity was observed (*I*^2^ = 95%), a random-effects model was used. In the plane rectangular coordinate system, the forest plot takes a vertical invalid line (scale of abscissa is 0) as the center, describes the effect quantity and 95% CI of each study by using multiple line segments parallel to the horizontal axis, and describes the effect quantity and confidence interval of multiple studies by using a diamond. CHFS, Cochin hand function scale; MSCs, mesenchymal stem cells; CI, confidence interval; SMD, standardized mean difference

CHSF is a questionnaire assessment for the extent of hand involvement in SSc patients, including 18 items: dressing, washing, other daily activities and working ability. [32, 33] The total score ranges from 0 (normal hand function) to 90 (severely impaired hand function). Furthermore, 2 articles compared CHFS changes before and after treatment with MSCs in SSc patients, and all the patients were administered autologous SVF via finger injection and followed up for about 24 weeks [21, 26]. A random-effects model was performed for high heterogeneity (*P* < 0.0001, *I*^2^ = 95%). Granel et al. [19] showed that the local injection of autologous SVF improve the grasping ability in SSc patients, and consolidated analysis suggested that there was no statistically significant difference in CHSF in SSc patients after 6 months of MSC treatment (MD = 9.05, 95% CI − 27.01 to 45.11, *P* = 0.62; Fig. 8).

## Pulmonary function

Only 1 study compared the changes in DLco and forced vital capacity (FVC) before and after treatment with MSCs in SSc patients. The average age of 14 patients with diffuse SSc was 37.4 years, and 3 of them were complicated with ILD. Patients with SSc-ILD showed no improvement in pulmonary conditions after treatment with glucocorticoids and cyclophosphamide. However, they received a single intravenous infusion of UC-MSCs (1 × 10^6^ cells/kg) in combination with plasma exchange and cyclophosphamide and showed significantly improved DLco and FVC of the 3 patients after 12 months (*P* < 0.05), suggesting that MSCs ameliorate pulmonary function damage in SSc-ILD patients.

## Safety

7/9 clinical studies reported the occurrence of AEs, including edema and pain at the injection site after lip injection of ADSCs in one study, which improved spontaneously or recovered in 1 week by symptomatic treatment. However, the number of AEs was unknown. A total of 16 cases of AEs, according to severity grade, were recorded in 114 SSc patients treated with MSCs in 6 studies, 15 cases of mild AEs, and 1 case of moderate AEs (skin infection at the orofacial injection site improved with oral antibiotics). No serious AEs occurred in all the patients with MSC therapy (Table 2).

## Publication bias

**Fig. 9:**
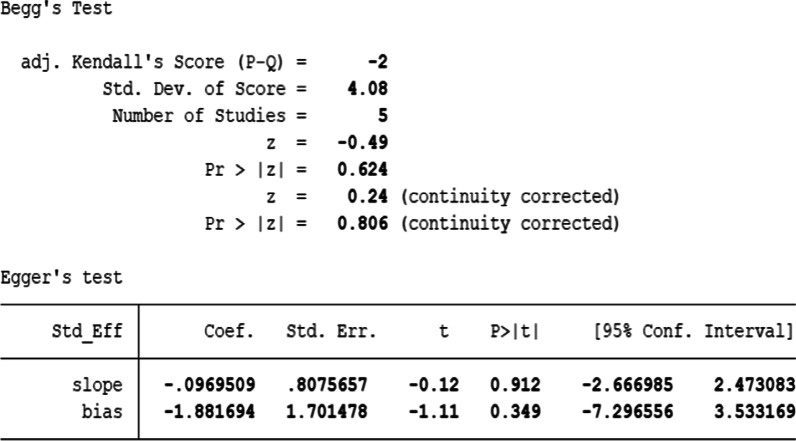
Publication bias. Funnel plot generated for the primary outcome using Begg's test (*P* = 0.806) and Egger's test (*P* = 0.349) suggested that there was no publication bias

In this meta-analysis, only mRSS primary outcome was recorded in 5 studies. Egger’s test (*P* = 0.349) and Begg’s test (*P* = 0.806) showed that the effect of MSCs from different sources on mRSS in SSc patients was symmetrical, suggesting no publication bias (Fig. 9).

## Discussion

To the best of our knowledge, this is the first systematic review and meta-analysis to carry out a comprehensive assessment of the efficacy and safety of MSCs in the treatment of SSc. Recently, stem cells have become a research hotspot in life sciences due to their self-renewal properties and multi-lineage differentiation potential. They are classified as totipotent, pluripotent, and specialized stem cells according to their differentiation potential. Compared to the totipotent and specialized stem cells, pluripotent stem cells have more advantages in less risk of teratoma formation and multiple differentiation potential. Among these, MSCs participated in the regulation of various signaling pathways by secreting cytokines, chemokines, growth factors, and extracellular vesicles, facilitating angiogenesis, anti-fibrosis, regeneration and immune regulation and becoming one of the potential therapeutic agents for SSc patients [9].

After repeated screening and checking, 9 self-controlled studies were included in this study, encompassing 133 SSc patients. The clinical application of mRSS is a vital technique for evaluating skin thickness in SSc patients. The current results demonstrated that MSCs improve mRSS and reduce the degree of skin thickening in patients with SSc, regardless of cell sources or follow-up periods. A remarkable decrease in mRSS in SSc patients was observed at 12 months of follow-up after treatment. Moreover, compared to traditional therapy (for example, conventional immunosuppressive agents or glucocorticoids), UC-MSCs (1–2 × 10^6^/kg, once a week, four times in total) combined with conventional therapy significantly reduced mRSS in SSc patients at 6 and 12 weeks with improved nail-fold microcirculation. In addition, functional organ damage in patients with SSc is closely related to the degree of fibrosis. Skin involvement often leads to orofacial skin thickening, lip skin wrinkling, and maxillofacial morphological changes, and even limited mouth opening, hand tendon contracture with dysfunction in severe cases. Herein, we found that MSCs promote skin elasticity and tightness at 3 months, reduce VAS and DU numbers at 6 months, decrease MHISS in SSc patients, and improve pulmonary function (DLco and FVC) of SSc-ILD patients at 12 months compared to pretreatment.

In addition, although RCS and CHSF were improved at 6 months after MSC treatment, no statistically significant difference was observed in the pooled analysis results for high heterogeneity between the two included studies. Notably, vascular endothelial growth factor (VEGF) and tumor growth factor-beta (TGF-β) play a critical role in angiogenesis. MSCs differentiate into dermal stromal cells and epithelial cells, secreting multiple cytokines (epidermal growth factor, stromal cell growth factor, and VEGF) to promote wound repair and angiogenesis [9]. The studies included in this meta-analysis revealed that, the number of giant capillaries and capillary density at the finger injection site of patients increased after local SVF injection, while malnourished capillaries decreased. Surprisingly, the non-injection site skin of SSc patients changed with varying degrees of alleviation; the mechanism needs further investigation.

Furthermore, the overall safety of SSc patients with MSC therapy was good without serious AEs. As mentioned above, 7/9 clinical studies reported AEs. A total of 16 AEs occurred in 114 SSc patients treated with MSCs (16/114), and the skin redness and swelling at the injection site were the most common AEs in patients, among which 1 case with local infection ameliorated after treatment with oral antibiotics, and 5 cases improved spontaneously. The other AEs, such as mild respiratory tract infection, diarrhea, or arthralgia, also recovered soon with symptomatic therapy. Another retrospective study recorded 1 case of tumor in 39 SSc patients with MSC therapy [34]. Currently, the consensuses on the effects of MSCs on tumors is poor [35–38]. Various factors may affect the potential of MSCs to differentiate into tumor cells, including donor factors (age), recipient environment, and complex regulatory mechanisms between the two cell types [39–41]. Therefore, MSCs are relatively safe for SSc treatment, and mild to moderate AEs occur in individual cases. Nonetheless, their safety needs to be confirmed by long-term follow-up in additional clinical trials.

In summary, MSC therapy improves the degree of skin thickening, lung function, and mouth opening, as well as relieves finger ulcers and pains in patients with SSc without severe AEs. MSCs or MSC combined with plasma and traditional medicine might be an effective and promising alternative for the treatment of SSc patients, especially those with severe disease, rapid progression of the disease, or refractory to conventional therapies. Alternatively, MSCs also decrease the titer or levels of serum antinuclear antibody and anti-Scl-70 antibody in SSc patients [28, 29]. However, the comprehensive mechanism and the overall validity and safety of MSCs in the clinical application need to be elucidated further. Hitherto, a large number of clinical trials of MSCs on SSc treatment have been registered. These are expected to provide strong clinical evidence for the unmet needs regarding MSC therapy, such as how to select MSCs from different tissue sources and donor types, infusion dose, frequency, survival time in vivo, and how to balance the advantages on combined therapy.

Nevertheless, the present study has some limitations. Firstly, there are a few RCTs on MSCs in SSc treatment. Since the included literature consisted of self-controlled studies, control group assessment was lacking, and clinical outcomes may be interfered with by the natural course of the disease. Secondly, only a few studies were included in the quantitative analysis. Publication bias may exist because the power of the funnel plot test is insufficient, and some studies with negative results could not be published. Thirdly, the unclear description of methods, hidden groups, and outcomes of the included literature may affect the final results. Finally, the period of MSC therapy and follow-up is insufficient due to the small numbers of patients, and data of long-term efficacy and safety with large sample sizes are essential.

## Conclusion

MSCs therapy improves the degree of skin thickening, lung function, and mouth opening and relieves finger ulcers and pain in patients with SSc without severe AEs. MSCs or MSCs combined with plasma and traditional medicine might be an effective and promising alternative for the treatment of SSc patients. However, the certainty of evidence ranged from moderate to very low.

## Data Availability

Not applicable.

## Abbreviations

SSc: Systemic sclerosis
COVID-19: Coronavirus disease 2019
ILD: Interstitial lung disease
AHSCT: Autologous hematopoietic stem cell transplantation
EULAR: European League Against Rheumatism
MSCs: Mesenchymal stem cells
PRISMA: Preferred Reporting Items for Systematic Reviews and Meta-analyses
GRADE: Grading of recommendations, assessment, development and evaluation
DU: Digital ulcer
mRSS: Modified Rodnan skin score
CRP: C-reactive protein
DLco: Diffusing capacity of the lung for carbon monoxide
MHISS: Mouth handicap in systemic sclerosis scale
CHFS: Cochin hand function scale
VAS: Visual analog scale
AEs: Adverse events
RCT: Randomized controlled trials
NOS: Newcastle–Ottawa scale
OR: Odds ratio
CI: Confidence interval
MD: Mean difference
ADSCs: Adipose-derived mesenchymal stem cells
SVF: Stromal vascular fraction
BMSCs: Bone marrow-derived MSCs
UC-MSCs: Umbilical cord MSCs
RCS: Raynaud’s condition score
PRP: Platelet-rich plasma
FVC: Forced vital capacity
VEGF: Vascular endothelial growth factor
TGF-β: Tumor growth factor-beta
